# Exploring Coracohumeral Ligament Elasticity Changes With Matrix Rhythm Therapy in Idiopathic Adhesive Capsulitis: A Case Report

**DOI:** 10.7759/cureus.66608

**Published:** 2024-08-10

**Authors:** Mrudula Sangaonkar, Gaurang D Baxi, Pallavi R Bhakaney, Tushar J Palekar, Rajesh S Kuber

**Affiliations:** 1 Physiotherapy, Dr. D. Y. Patil College of Physiotherapy, Dr. D. Y. Patil Vidyapeeth, Pune, IND; 2 Radiodiagnosis, Dr. D. Y. Patil Medical College, Hospital and Research Centre, Dr. D. Y. Patil Vidyapeeth, Pune, IND

**Keywords:** range of motion, pain management, matrix rhythm therapy, coracohumeral ligament, idiopathic adhesive capsulitis

## Abstract

Idiopathic adhesive capsulitis is a complex pathology that combines inflammation and tightness of the ligamentous and capsular structures in the shoulder joint. Using methods like mobilization, strengthening exercises, and stretching modalities, physiotherapy is still the mainstay in treating adhesive capsulitis. However, novel treatment modalities are being used in various musculoskeletal conditions. This case study investigates the effectiveness of matrix rhythm therapy (MRT) in treating a 42-year-old female patient with idiopathic adhesive capsulitis by examining the elasticity of the coracohumeral ligament (CHL). The patient received six sessions of MRT over two weeks, in addition to stretching exercises for the CHL and capsular tissues. The modality's effectiveness was recorded using a universal goniometer for measuring the range of motion, the Numeric Pain Rating Scale (NPRS) to measure pain, and elastography to measure CHL thickness. Significant improvement in pain and range of motion was noted. Additionally, ultrasonographic evaluations revealed a substantial reduction in thickness from 1.9 mm to 0.9 mm and in CHL shear ratio from 2.94 to 0.40. MRT has shown a beneficial effect on CHL elasticity, which helped in the case of idiopathic adhesive capsulitis, showing a possibility as a viable technique for addressing deeper tissue involvement in shoulder disorders.

## Introduction

Frozen shoulder, also referred to as adhesive capsulitis, with a prevalence of 3% to 5% in the general population, is a limiting shoulder condition marked by pain, stiffness, and a gradual loss of both active and passive range of motion [1]. For those who are affected, it limits functional activities and quality of life due to pain. Idiopathic adhesive capsulitis is a complex pathology that combines inflammation and tightness of the ligamentous and capsular structures in the shoulder joint, especially the coracohumeral ligament (CHL) [2]. The CHL is essential in limiting external rotation and abduction, which greatly adds to the functional impairment that adhesive capsulitis patients suffer [3]. Adhesive capsulitis is a common condition, but there are many traditional methods like manipulation under anesthesia and capsular release show improvements in function but are frequently linked to surgical complications [4]. Using physiotherapy modalities like therapeutic ultrasound, hot fomentation, interferential therapy, and manual therapy like mobilization, strengthening, and stretching exercises, physiotherapy is still the mainstay of treating adhesive capsulitis [5]. Dr. Ulrich Randoll's creation, matrix rhythm therapy (MRT), offers an innovative approach to addressing the underlying cellular and extracellular matrix dynamics in a range of musculoskeletal conditions. The therapy is based on the idea that each tissue and cell in the body has inherent oscillations that occur at frequencies between 8 Hz and 12 Hz [6]. These oscillatory frequencies can be disturbed by trauma and injury, which can result in discomfort, restricted movement, and impaired cellular function. The goal of MRT is to restore equilibrium to the extracellular environment, which in turn facilitates the restoration of impaired cellular functions and supports general health, pain management, and tissue regeneration [7]. The CHL becomes a crucial point to consider when addressing adhesive capsulitis. Research employing shear wave elastography has demonstrated that the CHL is more rigid in affected cases of adhesive capsulitis when compared with unaffected ones. This increased rigidity plays a major role in the range of motion restrictions, especially in external rotation and abduction [8]. Novel therapeutic approaches are necessary because there are few treatment modalities for adhesive capsulitis that specifically target tissue-specific pathologies. This case study explores the potential utility of MRT for treating idiopathic adhesive capsulitis patients' extensibility of the deeper tissues, specifically the CHL. By means of an extensive clinical assessment including range-of-motion measurements, ultrasonographic analysis, and pain assessments, our objective is to clarify the therapeutic effects of MRT on the CHL and, in turn, its impact on pain and shoulder function. This case study aims to provide insights into the effectiveness of MRT as a targeted intervention for the management of adhesive capsulitis by concentrating on the CHL and its elasticity.

## Case presentation

The patient in the present case study is a 42-year-old woman who has had limited range of motion in abduction and internal and external rotation with persistent shoulder pain for the past 18 months. The patient complained of being unable to lie on her right shoulder and experiencing pain when doing overhead tasks. Idiopathic adhesive capsulitis, which is characterized by pain, stiffness, and functional impairment, was diagnosed following a clinical evaluation, which showed active and passive movements restricted in capsular pattern, along with increased thickness of CHL assessed on ultrasonography. The patient was initially treated conventionally after diagnosis, which involved a one-month course of analgesics and anti-inflammatory medications, along with the use of hot fomentation. Because of severe pain and functional limitations, physiotherapy was recommended. According to the Numeric Pain Rating Scale (NPRS), the patient's clinical evaluation indicated pain levels of 4/10 while at rest and 8/10 when performing any activity. Using a universal goniometer (intraclass coefficient: 0.83-0.98), range of motion measurements revealed significant limitations: right shoulder flexion at 110 degrees, right shoulder abduction at 90 degrees, right internal rotation at 25 degrees, and right external rotation at 28 degrees (Table 1). The Shoulder Pain and Disability Index (SPADI) was used to measure functional disability.

**Figure 1:**
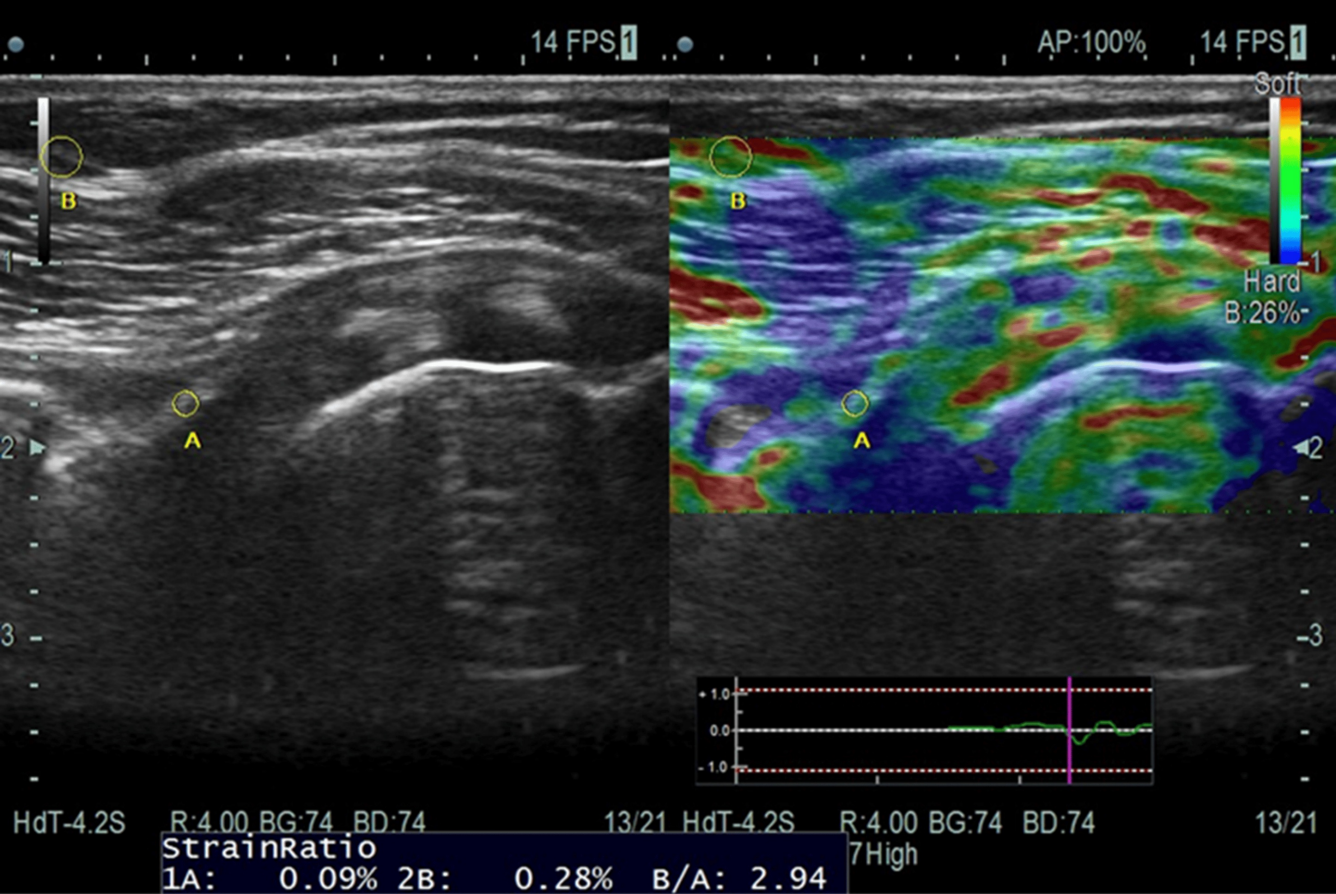
(A) Targeted tissue CHL; (B) subcutaneous fat at a distance of 1 cm Strain ratio: subcutaneous fat tissue strain/target CHL strain; CHL: coracohumeral ligament; cm: centimeter

The CHL was assessed using ultrasonography. Shear ratio and CHL thickness were measured using elastography. The initial ultrasonography results showed a CHL thickness of 1.9 mm and a shear ratio of 2.94 (Figure 1).

Owing to the patient's persistent symptoms and restricted reaction to traditional therapies, an MRT regimen was initiated for two weeks, in which six sessions were performed. The patient received targeted stretching exercises that targeted capsular and CHL mobility in addition to MRT. After the MRT sessions, a thorough reevaluation was carried out. The improvements seen in the range of motion were quite significant (Table 1).

**Table 1:** Pre-treatment and post-treatment range of motion of shoulder joint ^0^: degrees

	Active range of motion		Passive range of motion	
Shoulder range of motion (right)	Pre-treatment	Post-treatment	Pre-treatment	Post-treatment
Flexion	100^0^	160^0^	115^0^	160^0^
Abduction	90^0^	150^0^	100^0^	156^0^
Internal rotation	40^0^	65^0^	43^0^	68^0^
External rotation	20^0^	50^0^	24^0^	55^0^

Further, functional evaluation was done on specific outcome measures and was noted as pre-treatment and post-treatment (Table 2).

**Table 2:** Outcome measures showing effectiveness of MRT on various parameters CHL: coracohumeral ligament; SPADI: Shoulder Pain and Disability Index; mm: millimeters

Outcome measure	Pre-assessment	Post-assessment
Pain	8/10	2/10
CHL thickness	1.9 mm	0.9 mm
Stress-strain ratio	2.94	0.40
SPADI	79.9%	36.9%

## Discussion

Adhesive capsulitis symptoms, such as pain, stiffness, and restricted range of motion in the shoulder joint, limit the patient's performance. Conventional treatments have the potential to enhance performance; however, they might not be able to target deeper tissue involvement, like that observed in the CHL [9]. This discussion explores the transformative effect of MRT on CHL elasticity in idiopathic adhesive capsulitis.

Coracohumeral ligament in adhesive capsulitis

It is generally known that the CHL contributes to adhesive capsulitis and is essential to shoulder joint mechanics. Patients with frozen shoulders have limits in external rotation and abduction, and one of the main causes of these restrictions is increased stiffness of the CHL. Shear wave elastography reveals this stiffness, which emphasizes the necessity for focused therapies to address the particular pathophysiology within the ligament [9].

Matrix rhythm therapy mechanism

Based on the idea of cellular oscillatory rhythms, MRT offers a special method for both pain relief and tissue regeneration. By restoring the typical oscillation frequencies that have been disturbed by trauma or injury, the therapy hopes to support tissue regeneration and cellular activity. MRT ought to restore normal tissue resonance which in turn causes improved venous circulation as well as lymphatic circulation. By targeting the dynamics of the extracellular and cellular matrix, MRT may be able to impact tissue-specific disorders like adhesive capsulitis [10]. A study done by Tanna et al. (2024) compared the effect of MRT on radiation-induced trismus with the myofascial release technique and concluded that both treatments were equally effective; however, the MRT group showed better satisfaction [11]. The effects of MRT have also been studied in patients with chronic lower back pain by Özcan et al. (2021), where they found a positive effect of MRT on pain, disability, and quality of life [12].

Pain reduction and improved range of motion

The case study demonstrated that after undergoing MRT, there was a significant decrease in pain levels and a significant improvement in range of motion. The scores on the NPRS dropped from 8 to 2, signifying a notable reduction in pain. Assessments of the range of motion, such as flexion, abduction, external rotation, and internal rotation, showed significant progress. These results are consistent with the hypothesized mechanisms of MRT, which include improved tissue flexibility and pain reduction through decreased inflammation and increased blood circulation [13].

Ultrasonographic changes in the coracohumeral ligament

Quantitative measurements of the CHL's shear ratio and thickness were obtained using ultrasonographic examination. A stiffened ligament with a shear ratio of 2.94 and a thickness of 1.9 mm was shown by the pre-therapy measurements. These measurements showed a significant improvement following treatment, with the thickness falling to 0.9 mm and the shear ratio falling to 0.40. These ultrasonographic alterations imply that the flexibility and structural characteristics of the CHL are significantly affected by MRT. As contracted CHL restricts the external rotation and abduction, its extensibility will lead to improvements in external rotation and abduction. The result of the study shows improved ROM for the same [14].

Functional improvement

After treatment, functional disability as measured by the SPADI significantly decreased from 79.9 to 36.9, in addition to improvements in pain and range of motion. This improvement in functional outcomes suggests that the patient's everyday activities are significantly improved as a result of the beneficial changes in pain and range of motion.

Implications and future directions

The results of this case study highlight how MRT can be used to treat the unique pathophysiology of CHL in cases of idiopathic adhesive capsulitis. The potential of the therapy to modify the dynamics of the extracellular matrix and cells offers a focused and successful intervention. However, more investigation into the wider application of MRT in the treatment of adhesive capsulitis, along with bigger sample numbers and controlled trials, is necessary to validate these results.

## Conclusions

In conclusion, this case study demonstrates how MRT improved a patient's idiopathic adhesive capsulitis. Significant pain relief, increased range of motion, and improved elasticity of the CHL were all demonstrated by the therapy. These results imply that MRT is a potentially useful intervention for people with adhesive capsulitis and that it should be investigated further in more extensive clinical trials.
